# Changing public perceptions of alcohol, alcohol harms and alcohol policies: A multi‐methods study to develop novel framing approaches

**DOI:** 10.1111/add.16743

**Published:** 2024-12-23

**Authors:** Niamh Fitzgerald, Kathryn Angus, Rebecca Howell, Heather Labhart, James Morris, Laura Fenton, Nicholas Woodrow, Maria Castellina, Melissa Oldham, Claire Garnett, John Holmes, Jamie Brown, Rachel O'Donnell

**Affiliations:** ^1^ Institute for Social Marketing and Health University of Stirling Stirling UK; ^2^ Centre for Addictive Behaviours Research London South Bank University London UK; ^3^ Sheffield Centre for Health and Related Research University of Sheffield Sheffield UK; ^4^ FrameWorks UK London UK; ^5^ Tobacco and Alcohol Research Group University College London London UK; ^6^ School of Psychological Science University of Bristol Bristol UK

**Keywords:** alcohol, alcohol advocacy, alcohol industry, commercial determinants, communications, framing, policy advocacy, public health, public opinion, qualitative

## Abstract

**Background and aims:**

Public perceptions of alcohol and its related harms and policies are shaped by multiple discourses and can influence behaviour and policy support. As part of a FrameWorks‐informed project to test framing approaches to improve public understanding and support for evidence‐based alcohol policies in the UK, this research aimed to (i) summarise relevant evidence; (ii) compare how public understanding of alcohol harms differs from those of academic and charity experts; and (iii) develop novel framing approaches.

**Methods:**

(1) a literature review including systematic, scoping and targeted components to understand previous evidence on effective framing from behaviour change, UK alcohol policy and FrameWorks literatures; (2) comparison of public views of alcohol harms and policies from four focus groups (*n* = 20) with those of public health experts; (3) an iterative process involving workshops and stakeholder consultation to develop 12 novel framing approaches.

**Results:**

We found no previous study that directly tested framing approaches for alcohol policy advocacy. Our narrative summary of 35 studies found that explaining diverse harms may be important, whereas framing that engenders empathy, emphasises dependence or invokes a sense of crisis may be less effective. In focus groups, the public linked alcohol to pleasure/socialising, whilst understandings of harm focused on severe alcohol problems and individual deficits of biology or personality, with policy proposals focused mainly on treatment/support services. Public health experts highlighted more diverse harms and solutions, emphasising environmental and commercial causes. Comparison of public and expert views yielded six tasks for novel framing approaches to deepen public understanding. The team co‐developed initial framing ideas (*n* = 31), before finalising 12 narrative framing approaches based on values (*n* = 5), metaphors (*n* = 3) and explanation (*n* = 4).

**Conclusions:**

In the United Kingdom, public and expert understandings of alcoholrelated harms, causes and solutions differ. Along with prior evidence, these differences can inform novel framing approaches designed to deepen public understanding.

## INTRODUCTION

Alcohol harms place a huge burden on society globally including physical and mental ill‐health and premature death, violence and disorder, low productivity, relationship breakdown and child maltreatment [1, 2, 3]. The most effective policies to reduce harm involve higher prices for alcohol, restrictions on marketing and reduced availability [4, 5, 6], but comprehensive policies are rarely enacted, in part because of opposition by commercial stakeholders and neoliberal ideologies [7, 8, 9, 10, 11].

Public opinion of alcohol policies varies between nations and over time [12, 13], but studies often find that younger people, males and heavier alcohol consumers are less likely to support government intervention [14, 15, 16, 17, 18]. Studies often find majority support for most policies, although price or availability measures seem less popular than marketing controls or enforcement around alcohol sales [15, 16, 19, 20, 21]. Public knowledge and beliefs about alcohol are important influences on behaviour, stigma and efforts to cut down [22, 23, 24] as well as attitudes to government action. Knowledge that alcohol consumption causes cancer is associated with support for alcohol policies [21, 25, 26], whereas a few studies show policy support increasing following exposure to cancer warning labels [27] or campaigns explaining cancer risk [28]. Deliberative approaches to gauging public opinion, such as citizens' juries, have become popular, including in alcohol policy [29]. Public opinion influences policy decisions, alongside dominant ideas and ideologies [30, 31].

Underpinning this article is the idea that public opinion is grounded in ways of understanding or ‘framing’ alcohol as a policy problem, and that public opinion can be changed if exposed to new ways of framing. Put simply, framing is about the ideas we have about a topic and how we share them. It is a process of making sense of an issue, expressing that sense by naming selected features of the problem (and excluding others), and weaving them into a narrative (or story) [32, 33]. Individual members of the public often engage in framing unconsciously, and framing is accepted as central to policymaking [34, 35, 36]. Alcohol policy may be understood as an ‘intractable policy controversy’ [33] in which competing stakeholders engage in a battle of ideas, hoping that their ways of framing the problem and the policy solutions they favour, dominate thinking [34, 37]. Framing has been identified as important in United Kingdom (UK) and international alcohol policy processes including in pricing [38], licensing [39] and marketing [9, 40, 41] and more generally [42, 43, 44, 45, 46]. Alcohol industry actors deploy preferred framing approaches consistently over time in multiple channels [47, 48, 49, 50, 51, 52, 53], for example, emphasizing ‘responsible drinking’ to shift responsibility away from companies or governments and onto individuals and their choices [54, 55]. Framing alcohol policy as a broad, multi‐sectoral, public health issue that requires a whole‐population approach was considered crucial to enabling policymakers to seriously consider minimum unit pricing (MUP) (a policy creating a floor price, below which alcohol cannot legally be sold) in Scotland, and public health advocates intentionally presented alcohol policy in this way [11, 38].

As health stakeholders and charities seek to build recognition of and support for effective, evidence‐informed action to reduce alcohol‐related harms [44, 56], they must choose what information and concepts to include in public, media and political communications and how to combine them in a convincing narrative. In effect, they must engage in framing, but with little evidence to guide them on what framing approaches (or narratives) are most likely to achieve their goals. There is increasing interest in evidence‐based approaches to framing, with the FrameWorks Institute and FrameWorks UK (non‐profit communications research ‘sister’ organisations, hereafter FrameWorks for brevity) prominent in the field. Inspired in part by FrameWorks, Alcohol Change UK (ACUK, a leading alcohol harm prevention charity) commissioned a project to develop and test framing approaches for public communication on alcohol. As part of that project, this study aimed to: understand current evidence on what frames or framing approaches are most effective for deepening public understanding of alcohol‐related harms and building support for effective policies to reduce those harms; describe and contrast UK public and expert's (e.g. alcohol charities, public health academics) understandings of alcohol‐related harm, causes of, and solutions to such harm; and engage diverse UK alcohol experts and stakeholders in developing framing approaches designed to deepen public understanding and build support for effective policy.

## METHODS

Framing research has multiple disciplinary origins and inconsistent terminology, for example, ‘frame’ can mean ‘a package of ideas’, an argument or a metaphor [57]. In many policy analyses, ‘frames’ have been analysed as static, relatively narrow concepts that can be taxonomized, but Van Hulst and Yanow [32] helpfully distinguish ‘framing’ as a dynamic process of sense making and narrative, in which the roles of policy actors and the policy process itself are conveyed, not just features of the policy problem. In this study, we use the term ‘framing’ broadly in line with their dynamic conceptualisation: we sought to develop narrative‐based framing approaches, highlighting different aspects of alcohol harms as a policy issue and different ways of conveying these aspects.

Our study was conducted in three stages summarised below. Our methods were influenced by, but did not attempt to replicate, a FrameWorks study. Constrained resources and research team expertise resulted in several adaptations, for example, we used focus groups to identify public views on alcohol, whereas FrameWorks usually conduct one to one cognitive interviews to examine thinking patterns.

### Stage 1: Rapid review

**FIGURE 1:**
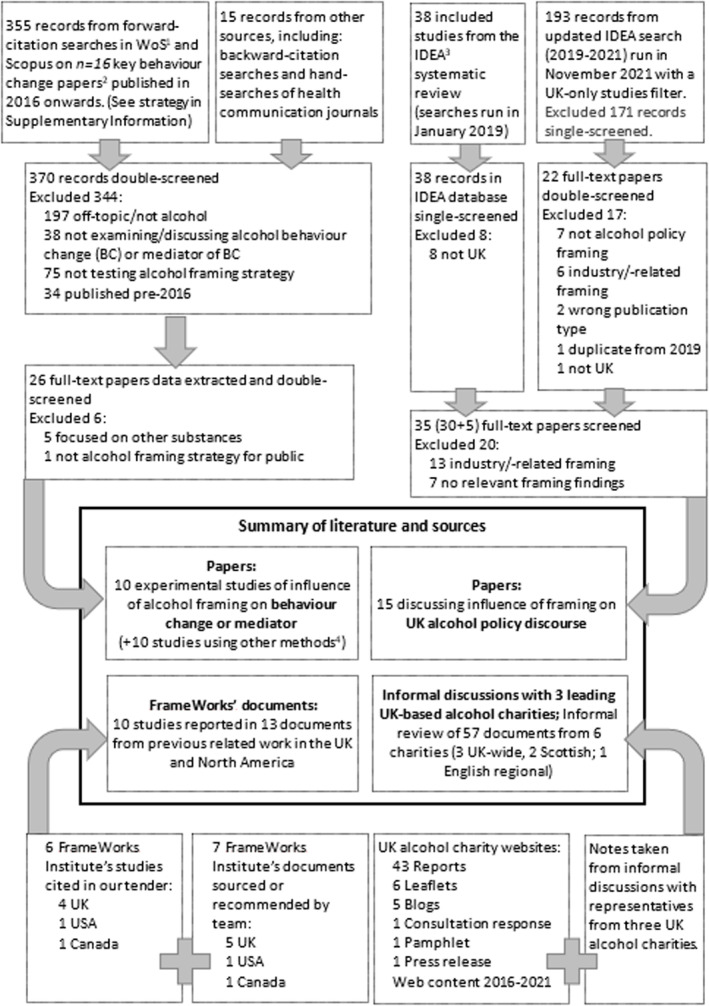
Rapid literature review.

First, we identified international experimental and qualitative research into how framing of alcohol issues influences behaviour change around alcohol from forward and backward citation searches on Web of Science and Scopus from a list of 16 initial publications known to the team as being relevant. This was supplemented with a hand‐search of health communication journals for alcohol studies (conducted in November and December 2021). Second, we used papers from a pre‐existing systematic review of framing of alcohol policy [58] for which we updated searches in November 2021 (Figure 1; search strategy in supplementary file). Papers were included if they focused on framing strategies used to advance public health policies for alcohol in the United Kingdom. Third, we consulted with FrameWorks and searched their websites (www.frameworksuk.org; www.frameworksinstitute.org) to identify FrameWorks research reports from any country on relevant social issues. We extracted and narratively summarised the findings from these three literatures on framing approaches found or thought to have been effective, organising them into five deductive categories (values, explanation, gain/loss conditions, issue frames and metaphors) selected pragmatically as common framing elements (Table 1) [59]. We conducted inductive coding of any other findings.

**TABLE 1:** Elements identified in the framing of social issues (definitions and examples).

Frame elements	Description	Example
Values	Values tap into people's shared commitments and priorities to make a case for why an issue matters and we should collectively work to address it.	'No child should go to bed hungry.'
Explanatory framing	Explanations are making causal relationships explicit, helping people to reason about potential solutions.	'Too often, affordable food options are high in sugar and fat—and healthier options are out of reach because of barriers like limited public transport. This leads to poorer health for people living in the areas most affected by poverty.'
Gain/loss conditions	A frame, which describes the impact of an issue in terms of what is gained or lost for society.	‘Inaction on children's health costs £5 million a year’ (a loss frame) vs. ‘action on children's health would save £5 million a year’ (a gain frame).
Issue frames	Issue frames foreground one dimension of a topic to establish what it is about. Changing an issue frame can dramatically affect public thinking and policy support	'Homelessness is a public health issue.'
Metaphors	Metaphors compare abstract, unfamiliar ideas to ones that are more straightforward and concrete. They can change the way a topic is understood.	'Our care system should be a scaffold of support around children'.

Separately, to identify examples of current framing practices of leading alcohol charities in the United Kingdom, we held informal discussions with three charities (1 English regional, 1 United Kingdom, and 1 Scottish) and drew on 57 documents from six charity websites (Figure 1). This was an informal review to deepen our own understanding and to find examples for illustrative purposes, and was not designed to systematically identify current practices.

### Stage 2: Public and expert views

The study was approved by the University of Stirling's General University Ethics Panel (4627). We recruited adults (18+) in the United Kingdom (*n* = 20) through a market research agency's in‐house research panel to take part in four on‐line semi‐structured focus groups lasting 1 hour. Participants were recruited for diversity (Table S1) in gender, age, social grade [59] and drinking frequency, offered a £30 incentive and gave on‐line consent. Groups were stratified by age and social grade and moderated by two researchers using a topic guide (Table S2) structured around the terms ‘alcohol’, ‘alcohol harms’, ‘causes of alcohol harms’ and ‘solutions to alcohol harms’. Each participant was asked to jot down and report their initial thoughts on these terms, before further discussion with the group. This minimalist questioning, ‘write and reveal’ method was designed to access individual default thinking and reduce in‐group effects. Audio‐recordings of each group were transcribed and anonymised. Inductive, open thematic coding was conducted manually for the four topics, reviewed by a second researcher and discussed with the team, before writing thematic summaries under the four headings with quotes. Both dominant and divergent views were coded and reported.

Separately, the research team, comprising public health experts on alcohol and ACUK colleagues took part in a workshop together, several meetings and an iterative written drafting process to develop a description of the nature and causes of, and solutions to alcohol harms in the United Kingdom as they perceived them, which we called an ‘expert story’ [60].

The expert story content was summarised under the same four headings as for the public focus groups above. Several team meetings were held with ACUK to compare the two summaries and discuss the clearest differences in understanding/views between the two groups. These discussions focused on identifying ‘tasks’ for novel framing approaches to shift public understanding closer to that of experts.

### Stage 3: Development of framing approaches

FrameWorks ran an on‐line workshop with the research team to generate framing ideas that could deliver on the identified tasks. These ideas were then discussed in a further workshop with alcohol policy stakeholders and a relevant government official; after which three attendees hosted further workshops with their colleagues. Throughout Stage 3, stakeholders provided feedback on the ideas via a shared on‐line document. After this consultation period, framing ideas were longlisted by discussion across the team. Three writing teams (J.M., J.B. and C.G.; J.H., N.W. and L.F.; N.F. and R.O.) expanded on selected ideas in long form text, which was further revised by N.F. and R.O. Feedback from ACUK informed a final selection and the final text of the framing approaches presented here.

## RESULTS

### Stage 1: Rapid literature review

The extracted documents are summarised in Figure 1. None of these literatures enabled definitive conclusions about effective framing for this study. The behaviour change literature yielded 10 experimental studies from multiple countries [23, 24, 61, 62, 63, 64, 65, 66, 67, 68] examining the impact of framing on mediators of behaviour change such as ‘problem recognition’ or stigmatising beliefs about people with alcohol problems. The UK alcohol policy literature (15 studies) included media [53, 69, 70, 71, 72, 73, 74] and documentary analyses [38, 75, 76, 77, 78], and stakeholder interviews [38, 70, 79, 80]. These studies were retrospective, mostly about MUP in Scotland. Importantly, they did not test the efficacy of framing approaches, but provided expert or author‐led hypotheses about helpful framing for public health policy progress. The 10 FrameWorks studies spanned three countries and used large scale qualitative and experimental methods to develop and test novel framing approaches to diverse social issues [81, 82, 83, 84, 85, 86, 87, 88, 89, 90, 91, 92, 93]. These did not examine attitudes to alcohol policy, but many of the gaps in public understanding in these social issues have parallels with alcohol (e.g. a focus on individual rather than structural solutions; feeling that harms affect only a minority group, see below).

Bearing in mind the limitations of the three literatures, we provide a brief summary of the most relevant findings for each element of framing, including illustrative examples of current framing by UK alcohol charities.

#### Values‐based framing

The idea that ‘children should be protected’ from alcohol harm was felt to be helpful in policy advocacy [69, 71], while emphasising the public's ‘right to know’ about the risks of alcohol was a feature of charity communications [94, 95]. Interdependence value frames (a collective responsibility to look out for others and collective benefits from doing so) were helpful in positively shifting public views of various social issues in several countries [81, 82, 83, 92, 93]. True stories about people bereaved through alcohol, which engender empathy, were reported as helpful by one alcohol charity. However, previous FrameWorks studies relating to mental health and addiction found that appealing to empathy‐based values was unproductive as it led to othering and a narrow focus on the individual and their behaviour, obscuring wider social and environmental causes [83, 93]. Another study found true stories to be effective at increasing the salience of an issue, but for them to increase support for structural solutions they need to be placed in a broader context and not play into existing stereotypes [92, 96].

#### Explanatory framing

Explaining how low‐cost alcohol leads to alcohol problems and the structural, population‐level causes of alcohol problems, without hard statistics, were felt helpful in building support for MUP in the United Kingdom [79, 80]. Explanations outlining how structural factors like poverty can drive mental ill‐health and foetal alcohol spectrum disorder, or how access to good transport and good education impact on physical health and obesity effectively improved public understanding [81, 83, 84, 87, 91].

#### Gain‐ or loss‐based framing

Outlining that diverse harms from alcohol could be reduced by MUP was common in United Kingdom advocacy [74, 78], with suggestions that benefits of MUP for specific groups should be discussed alongside population‐wide gains [69]. This was echoed in several FrameWorks studies in which framing focussing on societal gains alongside individual gains was effective [82, 83, 86, 88]. The economic costs of alcohol were commonly communicated by charities [97, 98], but this emphasis (e.g. on saving public money) was not found to be an effective approach for public communication in several FrameWorks studies [82, 85, 88, 92], perhaps because it could lead to fatalist thinking. In the case of obesity, it risked people believing that if the National Health Service is a ‘limited commodity’, patients whose ailments are perceived to be a result of their own choices could be a lower priority for treatment and could increase blame [89].

#### Issue‐based framing

This was the most widely discussed framing element across the different literatures. Framing of social issues in individual terms, for example, framing alcohol problems in terms of addiction or biology, is often unhelpful, because it focuses thinking away from structural causes and regulatory interventions [81, 82, 83, 84, 85] and is associated with negative stereotypes and stigmatization [68]. In contrast, framing using the concept of a ‘continuum’ of alcohol problems (emphasizing no clear boundaries between groups), can help people to recognise their drinking as problematic, in part by avoiding the need to identify as part of a stigmatised ‘other’ group of ‘problem drinkers’ [23, 24]. The policy literature hypothesises that if alcohol harm is framed as a cultural issue, it may be perceived as intractable [71], leading to fatalism, but that framing alcohol harms as a ‘public health’ issue may be more effective [11, 69].

#### Metaphor‐based framing

Metaphors can strengthen public understanding on social issues, for example, emphasising how the economy ‘restricts and restrains’ people in poverty was an effective metaphor for building public understanding of poverty [88]. UK alcohol charities sometimes used metaphor, for example, in crisis messaging about alcohol treatment services being ‘on their knees’ [99]. FrameWorks found crisis‐based metaphors ineffective for several social issues, as it led to fatalist thinking [83, 84, 85, 86, 87].

Beyond the above elements, FrameWorks studies suggest that providing clear reasons or motives for social change is important, as well as showing that change is achievable [81, 83, 86, 88, 89].

### Stage 2: Identifying the gap between public and expert views

Focus group participants focused primarily on positive effects of alcohol, ‘othering’ alcohol harms, and individual causes of and solutions to harm. Table 2 summarises their views with illustrative quotes including both common and divergent views. Their reported first thoughts about alcohol were almost universally positive, with a focus on socialising, sporting occasions, celebrations and stress relief. Negative issues were rarely spontaneously mentioned across the groups, until prompted to think about ‘alcohol harms’. Although the term ‘alcohol harms’ was unfamiliar, it brought to mind physical risks such as liver damage, drink‐driving or violence, as well as dependence, all mostly discussed as extreme effects affecting other people. They used metaphors when discussing dependence that emphasised a loss of control like ‘slippery slope’. Harms to people other than the drinker, impact on services, the economy or wider society were rarely mentioned.

**TABLE 2:** Expert and public views of alcohol, alcohol harms, causes of alcohol harms and solutions.

	Expert views	Public views	Quotes
Alcohol	Alcohol is inherently dangerous, being intoxicating, gradually addictive, and damaging to the brain and body. It is not like other foods/drinks and needs to be handled carefully by individuals and society.	Most participants talked about drinking alcohol as a positive and functional personal experience. They saw alcohol consumption as normal, widespread and expected. Very few mentioned negative impacts.	‘I pretty much drink when I'm socialising with friends or family and just see it as like yeah, something similar to a treat.’ (FG1, male) ‘Working through the week you look forward to your weekends and I guess having alcohol is a way to release any stress you are holding so you can kind of let go and have more fun than usual.’ (FG3, female) ‘I feel like any sort of big occasion, especially if you play sports, there's sort of a drinking culture around playing sports. Drinking and sports…like they go hand in hand together.’ (FG3, male)
Alcohol harms	Drinking alcohol leads to harms for many drinkers, not just a small minority, as well as their families, children and communities, workplaces, public services and society. Harms go beyond early death and ill‐health, to accidents, violence, suicides, domestic abuse, relationship breakdown, and lost days of work. The more alcohol people drink, as individuals and as a society, the greater the harms that are caused by alcohol.	The public noted the risk of embarrassment from ‘drinking to excess’, but otherwise focused on physical harms, primarily liver damage, violence and drink‐driving. Many thought of dependence, which was viewed as arising from a loss of control. Most harms were spoken about as only relevant to other people.	‘When you wake up the next day and you do not know what you have done the night before—the social harm where you could have done something embarrassing, you see all these messages on your phone, it can be really embarrassing.’ (FG1, male) ‘[Alcohol harms include] the critical illnesses like liver problems, that kind of stuff, accidents while you are drunk…Addiction, drink driving…kind of using it as a self‐harm tool as well.’ (FG3, female) ‘I think for many people it's a long slippery slope, some people do not actually realise they are becoming addicted…going from a couple of glasses of wine a night and then needing more to get that same hit.’ (FG2, female) ‘It can wreck families, fear of domestic abuse—dreading what kind of mood they'll be in when they come home, not just causing harm to the person whose drinking but also to their friends and family…People who are dependent and who drink too much are intoxicated all the time, are not aware of how they are harming themselves and those around them…not carrying out their parental duties.’ (FG2, female)
Causes of alcohol harm	Experts focused on structural and societal causes of greater alcohol consumption and related harms: Businesses making large profits from selling alcohol, giving them an incentive to normalise and promote alcohol consumption as essential, including by spending large budgets on advertising and marketing. social pressures and attitudes that are less accepting of or devalue choices not to drink alcohol.	Participants predominantly focused on individual causes of alcohol harms such as biology, choices, or personality, or drinking alcohol to cope with something else. The widespread availability of alcohol was also mentioned by a few participants; one group mentioned advertising.	‘Someone can have a (greater) potential for addiction; I think people's bodies can handle alcohol differently; I think it might be chemistry, a chemical thing, a chemical imbalance (in the body) that means people cannot stop.’ (FG2, male) ‘It's down to the individual to moderate it…you have got to take responsibility for your actions.’ (FG2, male) ‘Being alcoholic is a personal circumstance and is slightly different from people like myself that like to party at the weekend.’ (FG1, male) ‘I'd probably say with young guys it's a way of self‐medicating to deal with problems outside of their control, these people who do not want to go to the doctor cos their mental health's poor so they turn to alcohol – ‘I can get absolutely out of my face and I do not have to think about it’, until the next day when they feel worse and it's a never ending cycle.’ (FG1, male) ‘It's almost too accessible…you can ring Deliveroo now and get them to go down the local supermarket at 11 o'clock at night and bring a bottle of wine, or a bottle of vodka…10‐20 years ago you would not have had the choice.’ (FG4, female)
Solutions to alcohol harm	Experts emphasised that harms were not inevitable and could be reduced through effective policies to change culture including: better regulation of where and how alcohol is promoted and sold. removal of industry influence from alcohol policymaking.	Public participants tended to emphasise a need for: culture change and greater individual responsibility. better support services for people experiencing alcohol problems. The potential role of stronger policies regulating advertising, availability or pricing or the actions of alcohol companies were less commonly mentioned and attracted mixed support.	‘I think maybe the culture needs to change but it's not going to be an easy thing to change.’ (FG3, male) ‘I think just having more options of finding advice on how to deal with it [alcohol dependence] would help.’ (FG3, female) ‘The government also needs to fund the health and social care. I'm not so sure about the drink companies having a role, they have got to make a profit and nobody's making you want to go out and buy it, if there wasn't a demand for it the drink companies would not be producing it.’ (FG2, female) ‘… regardless of everything you have got to take responsibility for your actions, you cannot use excuses.’ (FG2, male) ‘I know my limitations, I'm in control of it and I can enjoy myself, why should my enjoyment be at sacrifice because some people you know are addicted to it?’ (FG3, female) ‘I think it's weird that the government allows you to put something in your body that can seriously harm you and you can end up in hospital. I feel there should be more restrictions, like there is with smoking.’ (FG1, male)

FG, Focus Group.

When asked about ‘causes of alcohol harm’, participants placed a strong focus on individual‐level deficits in personality, biology or behaviour (people drinking alcohol to cope) and sought to differentiate ‘other’ people as dependent on alcohol, who were viewed as different from themselves. Participants were keen not to directly stigmatise or vilify. They rarely discussed structural drivers of alcohol harms (such as commercial activities) apart from noting the widespread availability of alcohol. Regarding solutions, participants focused on downstream interventions like alcohol treatment services and funding for health care, and much less on (upstream) prevention. Individual agency and self‐control were seen as key mechanisms to avoid harm. Participants demonstrated ambivalence about culture change and about restrictions on alcohol advertising and promotion or days/hours of sale on the few occasions these were mentioned, being unsure about their efficacy or fairness. They were similarly ambivalent about the role or responsibilities of alcohol companies.

In contrast, public health experts conceptualised alcohol as an inherently risky and addictive substance that needed to be handled carefully by society. They focused on two main causes of alcohol harm: societal expectations and norms of alcohol consumption that exert pressure on people to drink, and a deficit of regulation to address widespread marketing and availability of alcohol, including cheap alcohol. They saw alcohol harms as being much more diverse than the public, affecting people, services and society beyond individual drinkers and viewed alcohol culture as a dynamic phenomenon, which could be shaped to reduce harms.

Given the contrast between public and expert views, we identified six key communication tasks for novel framing approaches (Table 3). In short, we aimed to build public understanding of alcohol as a toxic drug (task 1) that causes diverse harms for diverse people (task 2), as a result of marketing, policy deficits and commercial activities (tasks 3 and 4), while normalising choices not to drink alcohol (task 5) and creating a collective belief that harms can be reduced (task 6).

**TABLE 3:** Communication tasks for the novel framing.

Task 1: Build public understanding of alcohol as a toxic drug. Build recognition that alcohol is a drug, unlike most other types of food and drink and so needs to be handled more carefully. Build understanding of how alcohol negatively affects the body and brain.
Task 2: Build public understanding that alcohol causes diverse harms for a diverse range of people. Move the public from a narrow perception of harms relating to a small minority of ‘problem’ drinkers. Move the public toward understanding the diverse harms of alcohol for a large number of people drinking at a range of levels. Move the public toward greater understanding of negative impacts of alcohol on our economy, services and society as a whole.
Task 3: Move public understanding away individual‐focused explanations of harm, to commercial drivers and policy deficits. Move public thinking away from the idea of alcohol problems being the fault of individual choices or biology. Increase understanding of the role promotion, easy availability and cheap alcohol play in how much and how often we drink alcohol, and therefore in causing problems and harms. Build recognition that we are influenced more than we like to realise. Build public support for greater restrictions on the promotion, availability and affordability of alcohol.
Task 4: Build public recognition of the role of alcohol companies in driving alcohol harm and preventing effective action to reduce harm. Build public recognition of the role that big companies play in increasing alcohol consumption both directly and indirectly. Build awareness of big companies' reliance on heavy drinking for their profits, and their role in actively encouraging drinking (not just responding to demand). Increase recognition of the role of big companies in discouraging government action to reduce harms, to avoid barriers to selling more alcohol.
Task 5: Normalise and build public support for choices not to drink alcohol. Increase public recognition of societal pressures to drink alcohol and reflect on how well choices not to drink are currently accepted. Normalise choices not to drink and reduce the expectation and peer‐pressure to drink, especially in social settings. Counteract the myth that reducing alcohol consumption means diminishing life or that drinking alcohol is ever essential.
Task 6: Build a sense of collective efficacy that, together, we can prevent and reduce alcohol harms. Combat fatalism about the inevitability of current levels of harm. Instil a greater belief that societal and policy changes can make a positive difference to our lives.

### Stage 3: Development and consultation on potential framing approaches to address the differences found in stage 2

The workshop session generated 31 preliminary framing ideas, which were reduced to a final shortlist of 12 fully developed framing approaches (titles in Table 4; full text in supplementary file). The main considerations in drafting and shortlisting were the review findings (e.g. using narrative and explanation more than statistics, avoiding crisis messaging or a focus on economic costs, emphasising a continuum of alcohol problems rather than a focus on dependence, and using metaphor) and the input of stakeholders involved (e.g. ease of understanding and relatability, avoiding scaremongering/despair, and raising awareness of harms before focusing on solutions). The final shortlist consisted of values‐based, metaphor‐based and explanation‐based framing approaches.

**TABLE 4:** Titles of framing approaches developed in this study (see full text in supplementary file).

Category	Title
Values‐based	1 The truth is that alcohol is not essential to anything. 2 There are more harms from alcohol, of many different kinds, than we are told. 3 We can reduce harms from alcohol and enjoy life. 4 It is not fair that people suffer to make profit for big alcohol companies. 5 People should be free to make choices about alcohol without expectations or pressure from anyone else.
Metaphor‐based	6 When we drink alcohol, it is hard to stay safe in the shallows. 7 Alcohol is disguised as a ticket to happiness, hiding how harmful it truly is. 8 We can move alcohol away from centre‐stage in our lives without spoiling the show.
Explanation‐ based	9 Anyone who drinks alcohol can experience alcohol harms or problems. 10 The harms from alcohol come about for many reasons beyond individual choices or culture. 11 Alcohol causes a wider range of harms than we often recognise. 12 We now know that if we drink less alcohol, fewer people will suffer and die from cancer.

Five values‐based approaches included two focusing on ‘truth’. Frame 1 suggests that the truth about alcohol has been ‘twisted’ over time to make it seem essential to diverse social occasions, highlighting how advertising plays into this. Frame 2 outlines the lack of information for consumers on diverse alcohol harms, problematizes advertising, and emphasises the need for the public to be ‘told the truth’, including on labels. Frame 3, based on ‘ingenuity’, outlines that we have the knowledge and ability, as a society, to reduce alcohol harm. Frame 4, a ‘fairness’‐based approach, suggests that it is not fair that big alcohol companies are allowed to profit from customers suffering alcohol‐related harms. Frame 5 is based on the value of ‘freedom’ and questions the central role of alcohol in society and whether people are really free to choose whether to drink or not.

Three approaches used metaphors. Frame 6 uses a metaphor of shallow/deep water to illustrate the intoxicating and addictive effects of alcohol and lack of warning signs about the dangers. Frame 7 uses a ‘disguise’ metaphor to suggest that the harms of alcohol are hidden behind a disguise of ‘fancy packaging, slick slogans’, branding and marketing. Frame 8 suggests that alcohol is too often placed ‘centre stage’ in our lives and that it could be moved to the side ‘without spoiling the show’.

Four approaches were explanation‐based, and as these were generally much longer approaches, they have a sub‐structure that starts with ‘key message’, followed by ‘explanation’, ‘final consequence’ and ‘solution’, to aid digestibility. Frame 9 explains in simple terms that alcohol problems exist on a continuum and that many drinkers experience alcohol harms we do not recognise as problematic. Frame 10 explains structural and environmental causes of alcohol harms beyond individual choices or culture. Frame 11 explains a wide range of alcohol harms at different levels of alcohol consumption including pressure on services. Finally, Frame 12 focuses on explaining cancer risks from alcohol, as stakeholders reflected on evidence that such knowledge may have a powerful effect [21, 25, 26, 27, 28].

## DISCUSSION

This three‐stage study resulted in a set of framing approaches designed to inform communications to change public perceptions around alcohol. We identified substantial differences between public and expert understanding, with the public focusing on positive effects of alcohol, ‘othering’ alcohol harms and individual causes of harm, while experts focused on intrinsic and more diverse harms associated with alcohol as well as regulatory and commercial influences. Using insights gained from a rapid literature review and a stakeholder consultation, we developed 12 framing approaches with potential to enhance and deepen public understanding. The approaches provide explanations of diverse alcohol harms and structural drivers of harm, emphasise values sometimes seen as universal, such as ‘truth’ and ‘freedom’, focus responsibility away from individuals and onto commercial and government actors, and/or seek to de‐normalise alcohol consumption, all while using an inclusive fourth person point of view, and seeking to avoid crisis messaging.

Similar public views have been expressed in prior UK qualitative studies. One found that people tend to base their policy views on perceived impact on problematic ‘others’, particularly harmful or young drinkers [15] and were more negative toward alcohol regulation (pricing, availability) than educational approaches. Participants had mixed views on investment in treatment services. In a separate focus group study about MUP (*n* = 105), participants reported that they drank to be ‘social’, for down‐time or to party [100]. They were sceptical about the effectiveness of MUP fearing unintended consequences, again, focusing on its potential effect on others: those with dependence or experiencing homelessness [100]. These studies asked about named policies rather than inviting participants to share their thinking on alcohol more generally. It is likely that our ‘write and reveal’ methods, which gave them an opportunity to suggest solutions without specific prompting, came closer to accessing participant's individual default thinking, although less so than individual interviews.

The stark gap between public and expert views of alcohol and alcohol‐related harms has important implications for alcohol policy. A focus on personal responsibility and lack of control in public views may reflect industry success in establishing these ideas in discourse and thinking over several decades [51, 54, 55]. This way of thinking enables easy criticism of policies aimed at the whole population as being unfair or unnecessary, including opposition led by libertarian politicians, commentators, or media, which may reduce their appeal to policymakers. It also logically points to more downstream interventions such as alcohol treatment and support services, which do not prevent problems and which many people cannot or do not want to access. Our study has identified and applied diverse evidence and expert views to develop framing approaches designed specifically to address these sorts of gaps and limitations in current framing approaches. Our framing approaches were developed for use in general public communication, and efforts to target communication at specific population segments would likely have resulted in different approaches for different groups.

The framing approaches we developed are narratives that express how to make sense of an issue, naming and selecting certain features of the problem (harms, norms and costs), and in some cases, the actors involved (government and industry) and the policy process itself (lobbying). They include several features outlined in policy theory, particularly the Narrative Policy Framework [36]. Several frames portray characters (heroes, victims and villains, e.g. the alcohol industry as a villain hiding the truth from consumers in frame 2), which has been found to increase the influence of policy narratives [101, 102, 103]. Others broaden the ‘scope of conflict’ by emphasising diverse harms and causes of harm from alcohol beyond the individual drinker, to others, services and the economy. This content is typically used by interest groups that perceive that they are ‘losing’ on a policy issue [103, 104]. Many of our framing approaches also emphasise ‘causal mechanisms’, typically used to assign responsibility and blame [36], in this case putting responsibility for alcohol harm onto government and industry, most clearly frame 10.

Our work has implications for current communications practice in that it illustrates the challenge facing those seeking to advocate for action to reduce alcohol‐related harm. Some of our review findings support current framing practice (emphasis on protecting children, addressing stigma), however, other strategies, such as crisis messaging, are routinely used despite not being well supported by evidence. The most effective framing approaches may not be the most obvious or popular ones. The approaches developed here are based on prior evidence, empirical analysis of gaps in public understanding and input and review by experienced academic and advocacy professionals, but the approaches still need to be tested. In the meantime, it may be prudent to be guided by these approaches rather than using those without similar empirical underpinning.

### Strengths and limitations

The framing approaches outlined here have yet to be tested, and our findings should, therefore, be viewed as hypothesis‐generating. Although our development process was heavily influenced by FrameWorks thinking, we deviated from typical FrameWorks methods in some important respects. Notably, we ran focus groups with the public rather than individual interviews, and to mitigate the resulting possibility of in‐group effects, we developed bespoke questioning techniques. We also used FrameWorks literature on other social issues, the findings of which may differ from what works for alcohol.

An iterative process of co‐production of framing ideas with stakeholders and the involvement of a large multidisciplinary team from five institutions was a strength as was the close, constructive collaboration with ACUK throughout. The scope of the study was necessarily influenced by ACUK priorities [6, 105]. Participants recruited from market research panels may hold different views to the general UK population, who may in turn hold different views from those in other countries. Four focus groups provide a relatively small sample for assessing public views, although other UK studies have had similar findings.

### Implications for further research

We will explore and test public responses to these framing approaches qualitatively and quantitatively, with effective approaches informing a communications toolkit. Importantly, this will not recommend exact message wording, but focus on ideas and concepts in line with our understanding of framing as a dynamic act, rather than the use of static phrases. Further research could consider (1) the degree of take‐up of these frames in United Kingdom advocacy; (2) whether similar or different approaches might be developed (or work) outside of the United Kingdom; (3) whether any approaches identified as effective lead to changes in policy stakeholders' understandings or framing; (4) whether alcohol industry actors adjust their messaging in response to the use of novel framing; (5) how effective framing approaches compare to current framing practice for alcohol and other unhealthy commodities; and (6) which approaches work best with different sub‐populations.

## CONCLUSION

There is a substantial gulf between current public and expert understandings of alcohol harms, what causes harms and what policies or other changes could reduce or prevent them. This study brings an empirical approach, influenced by the work of FrameWorks, to facilitate bridging this gap. We used prior evidence, qualitative data and stakeholder co‐production to develop novel framing approaches, therefore going beyond reliance on the expertise of advocacy or communication professionals. We propose 12 narrative‐based framing approaches intended to strengthen public understanding of alcohol issues and increase support for effective policies. These approaches are likely to be more effective than some in common usage such as crisis messaging, but require further testing.

## AUTHOR CONTRIBUTIONS

**Niamh Fitzgerald:** Conceptualization (lead); data curation (equal); formal analysis (supporting); funding acquisition (lead); investigation (lead); methodology (lead); project administration (lead); visualization (supporting); writing—original draft (lead); writing‐review & editing (lead). **Kathryn Angus:** Conceptualization (equal); formal analysis (equal); writing‐review & editing (supporting). **Rebecca Howell:** Conceptualization (supporting); formal analysis (equal); writing‐review & editing (supporting). **Heather Labhart:** Conceptualization (supporting); formal analysis (supporting); writing‐review & editing (supporting). **James Morris:** Conceptualization (equal); data curation (equal); formal analysis (equal); funding acquisition (equal); investigation (equal); methodology (equal); writing‐review & editing (supporting). **Laura Fenton:** Conceptualization (equal); formal analysis (equal); writing‐review & editing (supporting). **Nicholas Woodrow:** Conceptualization (equal); formal analysis (equal); writing‐review & editing (supporting). **Maria Castellina:** Conceptualization (equal); methodology (equal); writing‐review & editing (equal). **Melissa Oldham:** Conceptualization (equal); writing‐review & editing (supporting). **Claire Garnett:** Conceptualization (equal); writing‐review & editing (supporting). **John Holmes:** Conceptualization (equal); supervision (equal); writing‐review & editing (lead). **Jamie Brown:** Conceptualization (equal); supervision (equal); writing‐review & editing (supporting). **Rachel O'Donnell:** Conceptualization (equal); formal analysis (equal); methodology (equal); supervision (equal); writing‐review & editing (supporting).

## DECLARATION OF INTERESTS

This project was funded entirely by Alcohol Change UK (ACUK), a leading UK charity, which works for a society free of alcohol‐related harm. ACUK was a valued and active partner in the reported research, and they had sight of findings as they emerged, influenced the development of tasks, the expert story and the final framing strategies presented here, but did not review or approve the text of this paper. C.G. and M.O. have done paid consultancy work for the behaviour change and lifestyle organization, ‘One Year No Beer’, providing fact checking for blog posts.

## Supporting information


**Table S1.** Sample profile by age, gender, social grade & drinking behaviour.
**Table S2.** Abbreviated Topic Guide for Public Focus Groups: Exploring Public Perceptions and Understandings of Alcohol.

## Data Availability

The literature review data extracts that support the findings of this study are available from the corresponding author upon reasonable request. The focus group data is not available for sharing due to privacy and ethical restrictions.
