# Supplementary material for: Changing public perceptions of alcohol, alcohol harms and alcohol policies: A multi‐methods study to develop novel framing approaches
Source: Addiction. 2024 Dec 23;120(4):655–68. doi: 10.1111/add.16743 (PMC11907333; doi:10.1111/add.16743)
Supplement: Supplementary file 1 — Table S1. Sample profile by age, gender, social grade & drinking behaviour. Table S2. Abbreviated Topic Guide for Public Focus Groups: Exploring Public Perceptions and Understandings of Alcohol. [file ADD-120-655-s001.docx]

**Supplementary Information**

**Policy literature – search strategy**

| **Sample Search**   1. **4 databases via Web of Science interface (KA, 13.1.16 and 21.11.21)**  - Science Citation Index Expanded (SCI-EXPANDED) --1900-present - Social Sciences Citation Index (SSCI) --1900-present - Arts & Humanities Citation Index (A&HCI) --1975-present - Emerging Sources Citation Index (ESCI) --2015-present     *Limited to: English language, peer-reviewed articles*   1. TS=(alcohol*) 2. TS=(policy OR policies OR policymaker$ OR policy-maker$) 3. AD=(policy OR policies) 4. SO=(communicat* OR critical* OR cultur* OR media OR policy* OR policies OR politic*) 5. #4 OR #3 OR #2 6. TS=((analys* OR synthesi*) NEAR/5 (discourse OR media OR document* OR content$ OR critical* OR framing OR frame* OR narrativ* OR descriptiv* OR text* OR thematic* OR stakeholder$ OR qualitative*)) 7. TS=((research) NEAR/3 (ethnograph* OR qualitativ* OR quantitativ*)) 8. TS=("case study" OR "case studies" OR map) 9. TS=("mass media" OR newspaper$ OR magazine$ OR televis* OR radio OR document* OR publication$ OR consultation$ OR submission$ OR debat* OR portray* OR discourse* OR representation$ OR coverage) 10. TS=((vested OR corporation$ OR corporate OR business OR industr*) NEAR/2 (interest*)) 11. TS=(stakeholder$ OR government* OR spokes* OR lobby* OR advoca* OR "think tank" OR critic$) 12. #6 OR #7 OR #8 OR #9 OR #10 OR #11 13. #1 AND #5 AND #12   KEY: TS Topic (title, abstract, keywords); AD Address; SO Publication Name; NEAR/x finds records where the terms joined by the operator are within a specified number of words (x) of each other; (*) any group of characters, including no character; ($) zero or one character.     1. **A similar search was run on 9 databases via EBSCOHost interface (KA, 13.1.16, 21.11.21):**  - Business Source Complete - Communication Source - Criminal Justice Abstracts with Full Text - Health Source: Nursing/Academic Edition - International Political Science Abstracts - MEDLINE - Political Science Complete - Public Affairs Index - SocINDEX with Full Text |
| --- |

**Table S1: Sample profile by age, gender, social grade & drinking behaviour**

| Group | **Age** | | **Gender** | | **Social grade** | | | | | | **Drinking behaviour (how often drink nowadays)*** | | | | | |
| --- | --- | --- | --- | --- | --- | --- | --- | --- | --- | --- | --- | --- | --- | --- | --- | --- |
|  | **Range** | **Mean** | **F** | **M** | **A** | **B** | **C1** | **C2** | **D** | **E** | **Never** | **x1 mnth or less** | **x1 mnth to x1 wk** | **x1 or x2 a wk** | **x3 to x5 a wk** | **More than x5 a wk** |
| G1 | 21-25 | 23.0 | 2 | 3 | - | 2 | 3 | - | - | - | 1 | 2 | - | 2 | - | - |
| G2 | 41-64 | 49.8 | 4 | 1 | - | - | - | 2 | 2 | 1 | - | 1 | - | 3 | 1 | - |
| G3 | 18-25 | 22.0 | 3 | 2 | - | - | - | 1 | 3 | 1 | - | 1 | 1 | 2 | 1 | - |
| G4 | 44-61 | 53.0 | 3 | 2 | - | 2 | 3 | - | - | - | - | - | 1 | 1 | 2 | 1 |
| All | 18-25  41-64 | 22.5  51.4 | 12 | 8 | - | 4 | 6 | 3 | 5 | 2 | 1 | 4 | 2 | 8 | 4 | 1 |

**Table S2: Abbreviated Topic Guide for Public Focus Groups: Exploring Public Perceptions and Understandings of Alcohol**

| **Introduction** | - Welcome and Study aims - Ground rules for focus group discussion - Outline session plan - Introductions |
| --- | --- |
| **1. Activity 1** | **What comes to mind when you think of the words ‘alcohol and drinking’?**   - Both good and bad things – no right or wrong answer, just whatever comes to mind. Please write down a few words or phrases that best describe your feelings about alcohol and drinking – it doesn’t need to be well written, so long as you can read it!   [2-3 minutes for activity]   - Can you please read out your thoughts (one at a time) – don’t change your responses based on what other people have put.      - [researcher to ask follow-up questions]   - Can you tell me a bit more about what you mean by x?   - Can you give me an example of y?   - Can you describe the kind of situation where z might happen?   [group discussion after everyone has spoken]   - As a group, what do you all think of what has been discussed? (explore similarities/differences, anything important that we might have missed etc) - Where do these understanding come from? (i.e. news, social media, personal experience etc) |
| **2. Activity 2** | **What comes to mind when you think of the words ‘alcohol harms’**   - Please write down a few lines about anything you think of - no right or wrong answer, just write down whatever comes to mind. For example:   - what do you associate with the term?   - what does the term mean to you (if anything)?   - Have you ever come across the term before – in what context have you heard it / would you imagine it being used?   [2-3 minutes for activity]  - Can you please read out your thoughts (one at a time) – don’t change your responses based on what other people have put.   - [researcher to ask follow-up questions]   - Can you tell me a bit more about what you mean by x?   - Can you give me an example of y?   - Can you describe the kind of situation where z might happen?   [group discussion after everyone has spoken]: what are the links with what you said about alcohol in Activity 1? |
| **3. Follow up:** | **What do you think are the causes of ‘alcohol harm’?**   - You said that ‘alcohol harm’ means x. - What do you think causes x? - Why do you think people behave in that way/chose to do that?   **What are potential solutions to alcohol harm?**   - You said that this kind of alcohol harm was caused by x, what do you think are the solutions to or what kind of actions might usefully address x)   (For each harm) **Who do you think is best placed to support these actions and who should be responsible for making these changes?**   - - government, local authorities, police, schools, parents, publicans, licensed trade, shops owners, supermarkets, alcohol producers, the public, drinkers etc |
| **4. Close** | - Thank participants for taking part, acknowledge that this can be a challenging topic to speak about - Provide debrief and contact information for researchers / supporting organisations - Provide participants with an opportunity to ask any questions, add anything we have missed or raise any points (both to the group, and directly to research team) |

**Full text of 12 framing approaches shortlisted to be taken forward for testing.**

**Values-based framing approaches**

1. **The truth is that alcohol is not essential to anything.**

- Truth matters. But, right now, most of us don’t know the truth about alcohol because it has become twisted over time.
- Many of us think about alcohol as being essential or central to socialising or relaxation or parties or celebrations.
- Big alcohol companies have spent billions on advertising and millions lobbying Government to keep us in the dark about the harms of alcohol, on fancy adverts to tell us we should reward ourselves with alcohol, and to promote the idea that we are not properly celebrating or socialising unless we’re drinking alcohol.
- We rarely question this but somehow we’ve lost sight of the truth that we can enjoy all of those things with less or no alcohol.
- When we put alcohol at the centre of those occasions, we can easily lose sight of what’s truly essential: being with people we have fun with, people we love, looking after ourselves, feeling able to really be ourselves.

1. **There are more harms from alcohol, of many different kinds, than we are told.**

- It’s important that we are told the truth about the products that are sold to us. Much of what we think we know about alcohol, is not actually true, and the reality is hidden from us.
- Drinking red wine is not good for our health, and the harms from alcohol go far beyond dependence and liver disease. But this is not what we are told in the media, on alcohol labels, or by the companies selling alcohol.
- Many of us think that alcohol is only harmful for other people, who drink more than we do. The truth is that for all of us who drink, cutting down would reduce our risk of cancer.
- Most people who cut down on alcohol sleep better, lose weight, have more energy and generally feel better. Yet most alcohol products don’t have ingredients or calories on the labels, and companies spend millions lobbying to keep it that way.
- Alcohol adverts have multi-million pound budgets to link drinking to friendship, sports and glamour. In reality, alcohol causes anxiety and depression but we’re not told that.
- It’s time we were told the truth so we can choose for ourselves.

1. **We can reduce harms from alcohol and enjoy life.**

- In Britain, we haven’t always drunk as much alcohol as we do now and we don’t have to drink this much in future. Before the past few decades, we drank alcohol a lot less, and less often, than we do now.
- We don’t have to have so many people getting illnesses like high blood pressure and cancer from alcohol, having their lives cut short.
- We can have fewer people hurt, annoyed, or worried by someone else’s drinking. We can also have fewer hangovers, sluggish days or missed evenings.
- We know how to fix this.
- We can change how we talk about alcohol to get a better balance. We can make alcohol less central to our lives and society.
- We can better manage how and where and when alcohol is promoted and sold, to make it easier to cut down or not drink, when we want to. We can do all this without changing who we are or how much we enjoy our lives.

1. **Its not fair that people suffer to make profit for big alcohol companies.**

- People shouldn’t suffer to make profit for big companies. Unlike other companies, the profits made by alcohol companies come at a high price for their customers, and the product they are selling is a toxic, gradually addictive drug.
- Companies spend millions on slick adverts and sponsorship deals to sell the idea of alcohol as a rewarding, fun or glamourous drink. They encourage and remind us to drink alcohol on every possible occasion.
- These companies need lots of us drinking at levels that are harming us to keep their profits high. They need us drinking at levels that lead to more ambulance call-outs, longer waits at A&E, and more crime in homes and on the streets They need us ruining our sleep, risking our health, and messing up our mornings, to make their money.
- The companies know this, but it would be bad for business to admit it. Instead, they spend vast sums of money making us think that all alcohol problems are the fault of ‘irresponsible’ people, not irresponsible companies.
- They lobby politicians to continue to be allowed to regulate their own advertising and to be able to sell alcohol in more places and around the clock. They pressure Governments to avoid being forced to put calorie or health information on their labels.
- Its not fair that they are allowed to do all this to keep their profits high, no matter what harms come to the people who drink the products they sell.

1. **People should be free to make choices about alcohol without expectations or pressure from anyone else.**

- People should be free to choose when they want to drink alcohol and when they don’t, but it often doesn’t feel that way.
- Alcohol is everywhere. We constantly see messages and pictures that suggest we need alcohol to really celebrate, relax and have fun. We’re told that drinking alcohol must be part of every occasion – from parties and weddings, to hotel stays and hairdressers, or even simple meals at home.
- This feels so familiar that we rarely question it anymore – it’s as if most of us go along with the idea that drinking is normal or even essential, and that not drinking is somehow wrong. If someone says they’re not drinking because they’re driving, we say ‘leave the car’; if they’re just having a night off, its ‘come on, have a drink’.
- Alcohol is a toxic, carcinogenic, gradually addictive drug, but we have set up our society in a way that pushes it on everyone. We make it harder for people not to drink. That’s not freedom. We can do better. People should be free to decide whether or not to drink alcohol without expectations from anyone else.

**Metaphor-based framing approaches**

1. **When we drink alcohol, its hard to stay safe in the shallows.**

- When we drink alcohol, it’s hard to stay in the shallows. We’re surrounded by advertising, special offers, and social pressures that tempt us to go deeper – telling us to “come on in, the alcohol’s fine”.
- One drink makes it harder not to have another, and it’s easy to get caught in the current.
- The deeper you go, the harder it is to drag yourself out. The water gradually gets more dangerous, with hidden harms beneath the surface and strong currents. Some of us will manage to clamber out unscathed, but we all know friends or family who have been hurt or out of their depth along the way.
- We need to prevent this happening.
- We need to stop alcohol adverts trying to persuade us to get in deeper.
- We need to stop the strong, super-cheap booze in every supermarket and corner shop that makes the current faster.
- We need clear warning signs, telling us of the dangers, before we get in.
- And we need to look out for each other, helping each other have fun without getting smashed by the swell or being dragged out to sea.

1. **Alcohol is disguised as a ticket to happiness, hiding how harmful it truly is.**

- From greeting cards to billboards, on supermarket shelves and bars, from fancy packaging to clever adverts, sponsorship deals and drinks promotions, alcohol is disguised as the best ticket to relaxation and fun.
- Behind the fancy packaging, slick slogans, and promised pleasure lies a silent killer in disguise. The fancy branding disguises the reality that alcohol is a toxic, addictive drug called ethanol that affects our bodies and our brain.
- Alcohol plays a role in more than 60 medical conditions, including stomach, bowel and breast cancers, high blood pressure, anxiety and depression. It causes more deaths and illness in younger people (15-49 year olds) than smoking.
- Alcohol companies pay big money to attach their brands to the things we love, whether its football, music, or friendship, so that the love we feel for the things we treasure in life rubs off on their brands. They are selling us alcohol and disguising all the harms it causes because they don’t want us to know the reality.
- We can quite happily make our own decisions about drinking alcohol or not, without allowing marketing to sell us a lie.

*Additional points:*

- Alcohol is dressed up as a treat, with no downside, but whatever the labelling, packaging, and adverts, it’s still the same alcohol underneath.
- Whether you drink it in beer, wine or spirits, ethanol is the ‘alcohol’ that changes how we feel as we drink, and that some of us get addicted to.
- Even if you don’t know someone who is addicted to alcohol, you’ll certainly know friends, loved ones and colleagues who have been harmed by alcohol, whether they know it or not.

1. **We can move alcohol away from centre stage in our lives without spoiling the show.**

- Alcohol too often takes centre stage. It is cleverly promoted as a hero at the heart of our lives.
- Family events, celebrations, friendships, meals, sport - alcohol is always expected, often seen as front and central to the whole experience.
- But alcohol isn’t the true star in our lives – get-togethers would be nothing without friends and loved ones; we don’t need advertising hoardings to enjoy a football match; we can cheer life’s wins without alcohol taking over the show.
- Too often alcohol is the diva that hoards the limelight, casting shade on what’s really important to us. Too often it overstays its welcome, stealing our mornings or messing up our plans.
- We can move the spotlight away from alcohol without spoiling the show.

**Explanation-based framing approaches**

1. **Anyone who drinks alcohol can experience alcohol harms or problems.**

**Key Message:** We often think that alcohol problems are only something that other people have. But in fact, there’s no clear line we can draw between people with alcohol ‘problems’ and anyone else who drinks alcohol.

**Explanation:**

- When it comes to drinking alcohol, many of us draw lines that say ‘my drinking is fine’ but ‘their drinking is a problem’. We do this to feel comfortable about our own choices, but really problems from alcohol can happen to anyone at any level of drinking.
- In terms of health problems, drinking very small amounts of alcohol is low risk, but still makes us more likely to get certain cancers.
  - Liver damage doesn’t just happen in people who have serious alcohol problems. Liver damage builds up slowly over time, without signs or symptoms until the damage gets pretty bad.
  - When we get anxious or depressed, drinking alcohol usually make things worse, often without us realising.
  - We don’t have to be drinking all the time to get high blood pressure from alcohol.
  - All of these problems affect our health and happiness and put pressure on our health services.
- Many of us experience other harms from alcohol that we don’t think of as ‘drinking problems’. We drink more than we meant to. We sleep badly. We feel rubbish the morning after. We get nothing done that we had planned. Sometimes drinking causes tension with family or friends.

**Final Consequence:** When we only notice the most severe alcohol problems and harms, it stops us recognising how we might all benefit from reducing how much we drink. It stops us asking the government to take effective action to reduce these harms that affect us all.

**Solution:** We can manage alcohol better as a society: people need better information on labels and adverts, proper restrictions on advertising and marketing, and better help to change their own drinking. We can reduce the harms from alcohol to benefit us all.

1. **The harms from alcohol come about for many reasons beyond individual choices or culture.**

**Key Message:** The high levels of drinking and alcohol harms we experience in the UK have come about for many reasons that go far beyond our individual choices or culture.

**Explanation:**

- Our choices and culture are hugely influenced by how we manage alcohol as a society –how we allow alcohol to be marketed and sold and how we talk about alcohol to each other.
- This environment strongly shapes our beliefs and attitudes towards drinking alcohol throughout our lives.
  - Global billion-dollar alcohol companies advertise drinking alcohol as a ‘fun’ and harm free activity, and they lobby to keep selling cheap alcohol, wherever and whenever they can.
  - We are not told about the health harms from alcohol – the anxiety, depression, sleep problems, high blood pressure, cancers, stomach ulcers etc. There’s no mention of these on adverts or labels.
  - Most shops have alcohol on display, with attractive packaging and cheap deals calling to us to buy.
  - Young children see alcohol advertising, fancy packaging, and sponsorships giving them a one-sided view that alcohol is only ever positive, glamourous or fun.
- Many of us go along with these ideas. We rarely talk about the downsides of alcohol – the hangovers, the family tensions, the lost days. We go along with the idea that only other people ever have ‘alcohol problems’.
- Because we have this one-sided view, we don’t have good information to make choices. And of course alcohol itself affects our brains, making it harder to resist another drink and undermining our decisions.
- We all like to believe that we are making our own free choices, but in reality when alcohol is heavily promoted, more easily available and at cheaper prices, we buy and drink more of it. And when we do try to cut down or stop drinking, seeing alcohol advertised and available everywhere makes it even harder!

**Consequence**: Alcohol problems come about for many reasons beyond someone’s genes or choices. The society we have developed is saturated in alcohol: we need to recognise how that pushes us towards drinking more and more until some of us can’t stop by ourselves.

**Solution:** We need to stop blaming people whose drinking causes them problems and start asking the government to take effective action to change the environment: less promotion, reliable information on labels and adverts, and proper support services so that we all have what we need to reduce the harms from alcohol.

1. **Alcohol causes a wider range of harms than we often recognise.**

**Key Message:** There are a whole range of significant and serious harms caused by alcohol across society but these are often not recognised.

**Explanation:**

- **Even small amounts of alcohol pose a risk to our health**. As we drink more alcohol, or consume it more often, our physical and mental health risks also increase including from cancers, high blood pressure, stomach problems, anxiety, depression and difficulties sleeping.
- **The harms from alcohol are not just to drinkers**. We are all affected by how much alcohol is drunk in this country, but we often don’t see these harms. Our health and emergency services are under pressure dealing with more alcohol-related illnesses as well as people who are drunk and aggressive; so we wait longer for treatment. Our communities are harmed when drinking spills over into anti-social behaviour, violence, and car crashes.
- **When someone who drinks alcohol at high levels is part of our family, we can also experience a range of problems.** We may worry about them, find that they contribute less to the family, that they’re not always fully present or can be unreliable. We’re also more likely to suffer poverty or domestic violence.
- **Even at lower amounts of alcohol**, people miss work or are less productive because they’re ill or hungover and others have to pick up the slack; or drinking causes tension in relationships.
- **The more we drink, the more harms we experience**: domestic violence, money problems, harm to children, unemployment and homelessness.

**Final consequence:** There are so many harms from alcohol and together they make life/society worse for us all, affecting drinkers, families, friends, colleagues, and taking up a lot of time, effort, and money for services and communities.

**Solutions:** To reduce the harms from alcohol we need to take effective action now to reduce how much we drink across the country and provide quality support services for those of us who need help to cut down.

1. **We now know that if we drink less alcohol, fewer people will suffer and die from cancer.**

**Key Message:** In recent decades, we have learned a lot about how to prevent cancer. We now know that if we drink less alcohol, fewer people will get cancer.

**Explanation:**

- Alcohol damages the cells in our bodies and changes hormone levels. These effects make us more likely to get certain cancers including breast, liver, mouth, throat and bowel cancer.
- Drinking alcohol does not mean that someone will definitely get cancer, but it makes it more likely. If you don't drink alcohol, your risk of getting cancer from alcohol is zero.
- The more alcohol each of us drinks, the bigger the chance we will get cancer – from the very first drink. That’s because more alcohol means more damage to our cells and bigger hormone changes.
- When it comes to cancer, it doesn’t matter what kind of alcohol we drink (beer or wine or spirits) or how we spread out our alcohol over the week.
- Even a small reduction in how much alcohol we drink reduces our risk of getting cancer.

**Final consequence:** Alcohol causes many cases of cancer including breast, liver and bower cancer. If we drink less as a population, more of us will live longer without being affected by cancer.

**Solutions:** If we want more people to live longer lives with their loved ones, free from cancer, we need to take action now to reduce how much alcohol is consumed in the UK. Less advertising, better information and improved support for people are all effective ways to do this.
